# Collagenous endobronchial obliteration after chemoradiation followed by consolidation osimertinib: a case report

**DOI:** 10.3389/fonc.2026.1895328

**Published:** 2026-07-31

**Authors:** Seunghun Lee, Juwhan Choi, Baek-hui Kim, Hakyoung Kim, Sung Yong Lee

**Affiliations:** 1 Department of Internal Medicine, Korea University College of Medicine, Seoul, Republic of Korea; 2 Division of Pulmonary, Allergy, and Critical Care Medicine, Department of Internal Medicine, Korea University Guro Hospital, Korea University College of Medicine, Seoul, Republic of Korea; 3 Department of Pathology, Korea University Guro Hospital, Korea University College of Medicine, Seoul, Republic of Korea; 4 Department of Radiation Oncology, Korea University Guro Hospital, Korea University College of Medicine, Seoul, Republic of Korea

**Keywords:** case report, chemoradiotherapy, collagenous endobronchial obliteration, non-small cell lung cancer, osimertinib

## Abstract

**Introduction:**

Consolidation osimertinib after concurrent chemoradiation is a standard treatment for unresectable stage III *EGFR*-mutated NSCLC, but endobronchial toxicity has not been well described. To our knowledge, this is the first reported case of collagenous endobronchial obliteration after concurrent chemoradiation therapy and consolidation osimertinib.

**Case presentation:**

We report an 83-year-old woman who developed acute dyspnea and complete right-lung atelectasis 3 months after completion of concurrent chemoradiation therapy while receiving consolidation osimertinib. Bronchoscopy showed membranous occlusion of all three right lobar bronchi, and biopsy demonstrated dense collagenous tissue admixed with fibrin and mild inflammation. Systemic glucocorticoid therapy and temporary interruption of osimertinib led to initial clinical improvement, but the obliteration recurred during corticosteroid tapering.

**Discussion:**

Compared with previously reported cases, our case demonstrated a more advanced fibrotic process, together with extensive involvement of all three right lobar bronchi within the irradiated field. Notably, this fibrotic change developed earlier than is typical for radiation-induced fibrosis. While radiation-induced airway injury was the principal cause, the early onset raises the possibility that osimertinib also contributed by impairing epithelial repair through EGFR inhibition.

**Conclusions:**

Clinicians should be aware of the potential for collagenous endobronchial obliteration, particularly in patients with centrally located tumors receiving chemoradiation followed by consolidation osimertinib. Bronchoscopic evaluation may facilitate diagnosis, whereas systemic glucocorticoid therapy with gradual tapering and close monitoring may aid management in similar cases.

## Introduction

In the phase 3 LAURA trial, consolidation osimertinib after definitive concurrent chemoradiation therapy (CCRT) significantly improved progression-free survival in patients with unresectable stage III epidermal growth factor receptor (*EGFR*)-mutated non-small-cell lung cancer (NSCLC), establishing this approach as a new standard of care (1, 2). Whereas radiation pneumonitis and fibrosis are well-recognized toxicities of thoracic radiotherapy (2, 3), radiation-related endobronchial injury has received less attention, even though central airway involvement may have serious clinical consequences. Radiation-induced pseudomembranous tracheobronchitis has been described in patients treated with CCRT without EGFR tyrosine kinase inhibitor (TKI) therapy (4). However, complete obliteration of the bronchial lumen by collagenous material has not, to our knowledge, been previously reported in a patient treated with CCRT followed by consolidation osimertinib.

## Case presentation

**Figure 1:**
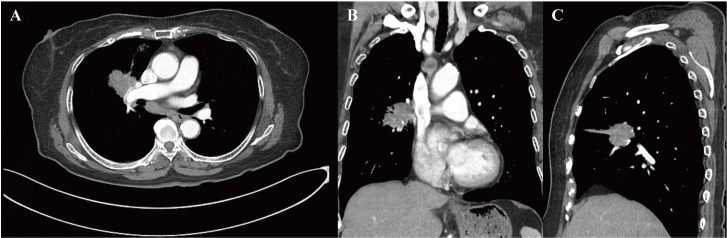
Baseline computed tomography (CT). **(A–C)** Baseline axial, coronal, and sagittal CT views revealing a 3.5-cm central mass in the right upper lobe.

An 83-year-old woman with hypertension and diabetes mellitus was diagnosed with right hilar adenocarcinoma (T2aN2bM0, *EGFR* L858R mutation). Baseline computed tomography (CT) showed a 3.5 cm centrally located right upper lobe mass (**Figure 1**). She underwent CCRT with three cycles of pemetrexed and carboplatin. Radiotherapy was delivered over the first and second months, and CCRT was completed in the second month. Radiographic assessment after CCRT demonstrated stable disease. Consolidation osimertinib (80 mg daily) was initiated approximately half a month after completion of CCRT.

**Figure 2:**
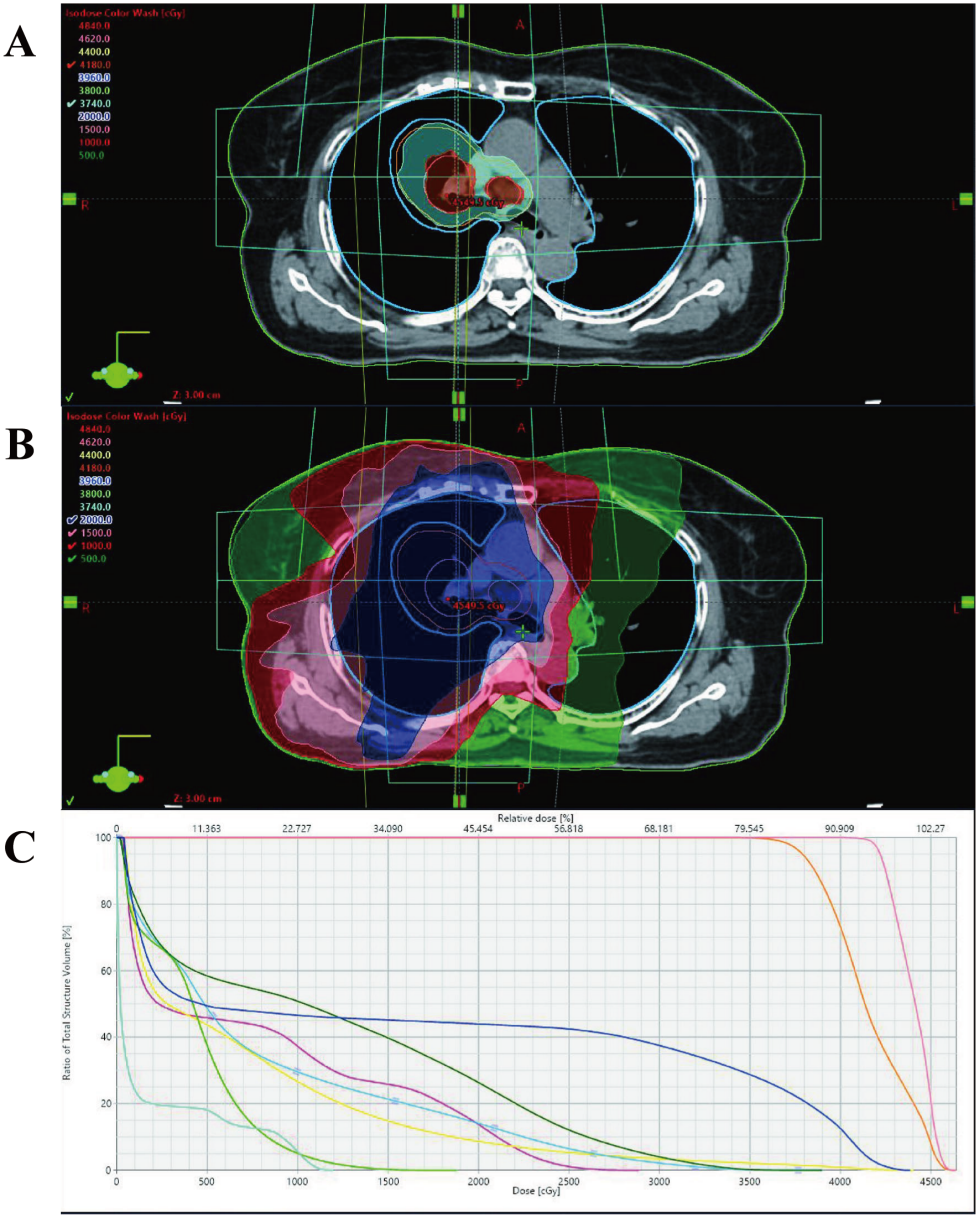
Radiation treatment plan. **(A)** Target-centered axial dose distribution demonstrating the 95% isodose lines for the gross tumor volume (GTV) and clinical target volume (CTV). The proximal bronchial tree is encompassed within the planning target volume (PTV). **(B)** Lung-centered axial dose distribution illustrating isodose lines corresponding to ≥ 5, 10, 15, and 20 Gy, with each dose level distinguished by different colors. **(C)** Corresponding dose–volume histogram demonstrating target coverage and sparing of surrounding organs at risk.

In accordance with our institutional protocols, the patient received intensity-modulated radiation therapy (IMRT) utilizing a simultaneous integrated boost (SIB) technique, delivering a total dose of 66/60 Gy across 30 fractions. For target delineation, the gross tumor volume (GTV) was outlined using the lung or mediastinal window setting. The internal target volume (ITV) was determined via four-dimensional CT to account for respiratory movement. The clinical target volume (CTV) was then generated by adding a 5 mm margin to the GTV-ITV in all directions, with adjustments made to protect adjacent normal anatomical structures. Subsequently, the planning target volume (PTV) was established by an additional 5 mm expansion of the CTV. The proximal bronchial tree, including the main and lobar bronchi, was completely located within the PTV, thereby receiving the full prescribed dose. Prescription guidelines dictated that at least 97% of the planned dose cover 95% of the PTV. The minimum and maximum doses delivered to 1 cc of the PTV were 95% and 107%, respectively. While institutional constraints required the lung V5, V20, and mean lung dose (MLD) to be maintained at ≤ 65%, ≤ 35%, and ≤ 20 Gy, respectively, this patient’s actual values were well within these limits, at 48.64% for V5, 14.08% for V20, and 8.19 Gy for MLD (**Figure 2**).

**Figure 3:**
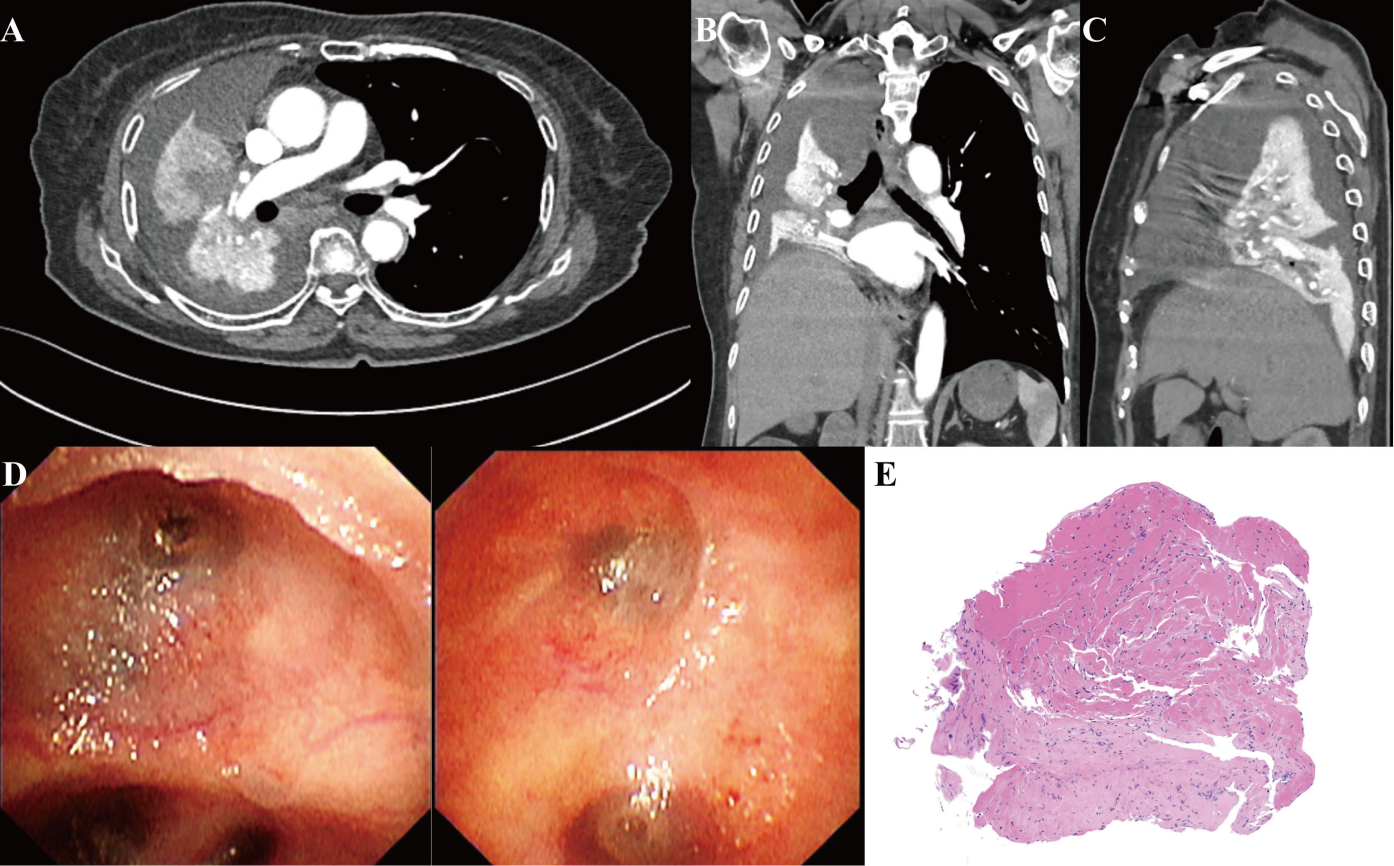
Clinical and histopathological findings of collagenous endobronchial obliteration. **(A–C)** Axial, coronal, and sagittal computed tomography (CT) views obtained during complete right-lung atelectasis, revealing occlusion of the right upper, middle, and lower lobar bronchi in the absence of an endobronchial mass. **(D)** Flexible bronchoscopy demonstrating web-like, avascular, whitish membranous structures sealing the orifices of the right lobar bronchi. **(E)** Histopathological evaluation (Masson’s trichrome stain) revealing dense collagen admixed with fibrin and mild inflammatory infiltrates.

Approximately three months after completion of CCRT, she presented with acute dyspnea and an oxygen saturation of 84% on room air. Chest radiography and CT images revealed total right-lung atelectasis with occlusion of the right upper, middle, and lower lobar bronchi, without evidence of an endobronchial mass. Flexible bronchoscopy showed web-like membranous structures causing complete occlusion of the right bronchial tree. The orifices of the right upper, middle, and lower lobar bronchi each appeared sealed by an avascular, whitish material. No significant endobronchial lesions were observed in the left bronchial tree, which lay outside the radiation field. Using biopsy forceps, the membranous structures were carefully perforated to re-establish airway patency. During bronchoscopy, endobronchial forceps biopsy of the obstructing tissue in the right lower lobar bronchus and bronchial washing were performed. Bacterial and acid-fast bacillus cultures of the bronchial washing yielded no growth, and the biopsy showed no evidence of malignancy. Histopathological examination with Masson’s trichrome staining confirmed dense collagenous tissue admixed with fibrin and mild inflammation (**Figure 3**).

**Figure 4:**
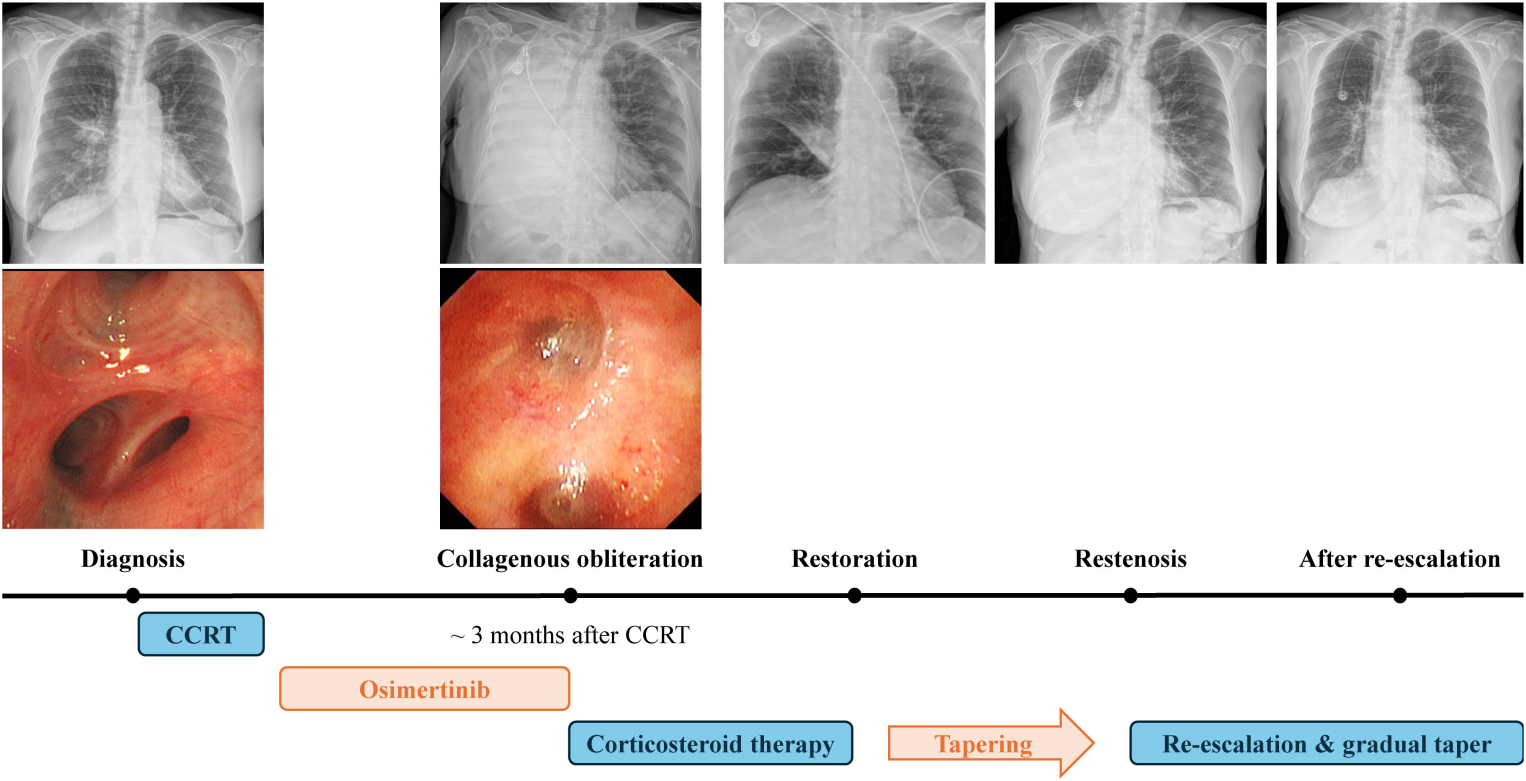
Clinical course with serial imaging and treatment timeline. Serial imaging and procedural findings are arranged chronologically from left to right. The chest radiographs (top) were obtained at diagnosis, at the time of complete right-lung atelectasis, at initial clinical improvement, recurrent atelectasis while corticosteroids were being tapered, and after corticosteroid re-escalation. Bronchoscopy showed preserved patency of the right lobar bronchi before concurrent chemoradiation therapy (CCRT) and, at the time of atelectasis, smooth, web-like collagenous occlusion of the right lobar bronchi with a silicone-like appearance. The timeline summarizes the clinical course, including CCRT, consolidation osimertinib, corticosteroid therapy, recurrent endobronchial obliteration during corticosteroid tapering, and subsequent corticosteroid re-escalation with gradual tapering. The initial endobronchial obliteration developed approximately 3 months after completion of CCRT during consolidation osimertinib.

Based on the bronchoscopic and histopathological findings, collagenous endobronchial obliteration was considered the most likely diagnosis. Accordingly, osimertinib was withheld, and intravenous methylprednisolone was started at 30 mg/day. After one month of systemic corticosteroid therapy, oxygen saturation recovered to 94% on room air, and follow-up chest radiography showed near-full resolution of the right-lung atelectasis. The clinical course, including serial chest radiographs and bronchoscopic findings, is shown in **Figure 4**. However, the obliteration recurred repeatedly during attempts at corticosteroid tapering, each recurrence requiring repeat bronchoscopic forceps perforation and corticosteroid dose re-escalation. Osimertinib had not been resumed, and its re-administration was being considered after completion of corticosteroid therapy. The patient remained on corticosteroids and was undergoing a gradual taper. The tumor showed no evidence of progression for approximately 6 months since initiation of consolidation osimertinib, consistent with stable disease.

## Discussion

Radiation pneumonitis has been the principal pulmonary toxicity reported with consolidation therapy after chemoradiation. The reported incidence of radiation pneumonitis of any grade was 20.2% with durvalumab in the PACIFIC trial and 48% with osimertinib in the LAURA trial, although differences in follow-up duration and reporting criteria limit direct comparison, and most cases in both trials were grade 1 or 2 (2, 5). Real-world studies in this consolidation setting have primarily reported pneumonitis rather than radiation pneumonitis. After consolidation durvalumab, a meta-analysis of real-world studies reported an all-grade pneumonitis incidence of 27% (grade ≥3, 8%) (6). A real-world cohort of consolidation osimertinib reported pneumonitis in 15% of patients (grade ≥3, 3%), with radiation pneumonitis explicitly excluded from the analysis (7). Risk factors for radiation pneumonitis have instead been characterized in real-world studies of the concurrent chemoradiotherapy setting. A meta-analysis identified lung V20 as an independent predictor of radiation pneumonitis (8), while a small study of elderly patients found that a MLD >20 Gy was associated with radiation pneumonitis (9). Other real-world studies, however, did not identify older age as an independent risk factor for acute toxicity following concurrent chemoradiotherapy (10, 11). Endobronchial complications were not reported in the pivotal trials and have not been systematically characterized in real-world studies. The collagenous obliteration observed in our patient may therefore represent an uncommon manifestation of airway toxicity following concurrent chemoradiotherapy and consolidation osimertinib.

Our case most closely resembles radiation-induced pseudomembranous tracheobronchitis, reported by Choi in two patients with bronchial obstruction after CCRT (4). Whereas the previously reported cases showed fibrin and inflammatory exudate without collagen, our case showed dense collagen admixed with fibrin. The progression of radiation-induced tissue damage follows a clear chronological sequence. It begins with an acute exudative phase marked by the accumulation of a fibrin-rich exudate, and this transitions into a late fibrotic phase characterized by progressive collagen deposition (12). Within this sequence, the fibrin present in both conditions points to a shared underlying process, whereas the additional collagen deposition in our case reflects progression to a more advanced fibrotic stage. Our case was further distinguished by its extent. All three right lobar bronchi, which lay within the high-dose radiation field, were simultaneously obliterated.

Radiation impairs endobronchial healing by reducing the airway basal/progenitor cell populations that support epithelial regeneration (13, 14). Concurrently, radiation induces the early release of profibrotic mediators, including transforming growth factor β (TGF-β), in irradiated tissue (15). TGF-β acts on fibroblasts in the bronchial submucosa, driving their differentiation into myofibroblasts that produce excessive extracellular matrix proteins, including collagen (3, 16). In the setting of concurrent epithelial loss, this unopposed profibrotic cascade may have culminated in the collagenous obliteration observed in our patient.

Osimertinib may have contributed to the unusually early fibrotic response observed in this case. Radiation-induced fibrosis typically develops from months to years after treatment, generally beginning 6 months or later after irradiation (17, 18). In the present case, however, dense collagenous obliteration was observed approximately 3 months after radiation. EGFR signaling is important for bronchial epithelial regeneration after injury, and pharmacologic inhibition of this pathway has been shown to suppress epithelial cell proliferation while leaving fibroblast proliferation unaffected (19). In our patient, consolidation osimertinib may have impaired epithelial repair in the irradiated bronchi, favoring fibroblast-mediated collagen deposition.

Radiation-induced vascular injury may also contribute to radiation-induced fibrosis. Endothelial cells are highly radiosensitive, and radiation-induced endothelial injury can result in microvascular occlusion and tissue hypoxia. Hypoxia stabilizes hypoxia-inducible factor-1α (HIF-1α), which promotes the expression of profibrotic mediators, including TGF-β1. Progressive collagen deposition further reduces vascularity, leading to gradual tissue ischemia that perpetuates fibrosis in a vicious cycle (20). Unlike normal wound healing, in which a balance between profibrotic and antifibrotic signaling promotes resolution of tissue repair, radiation-induced fibrogenesis has been described as “a wound that does not heal,” with persistent profibrotic signaling that perpetuates the wound-healing response (21). Such persistent fibrogenesis may also explain the recurrent endobronchial obliteration observed in our patient. Although the lesion initially responded to systemic corticosteroids, persistent profibrotic signaling and progressive collagen accumulation may have contributed to repeated restenosis during steroid tapering. These findings suggest that gradual corticosteroid tapering with close monitoring may be required in similar cases.

A strength of this report is the multimodal and longitudinal documentation of the clinical course, including radiologic imaging, bronchoscopy performed both before and after the onset of the obliteration, and histopathological confirmation of the diagnosis. The availability of the radiation treatment plan further allowed the obliterated bronchi to be correlated with the high-dose region, supporting a relationship between the complication and the irradiated field. This report also has limitations. The exact pathogenesis remains uncertain, and causality cannot be established from a single case. Nevertheless, radiation-induced airway injury was likely the principal driver of this complication, while osimertinib may have contributed to its unusually early fibrotic progression by impairing epithelial repair through EGFR inhibition. An additional limitation is the possibility that the biopsy specimen obtained during bronchoscopy may not have fully represented the obstructing material.

## Conclusion

As consolidation osimertinib after chemoradiation is increasingly used following the LAURA trial, clinicians should be aware of the possibility of collagenous endobronchial obliteration within the irradiated airway field, particularly in patients with centrally located tumors. During radiation treatment planning, the use of respiratory-gated radiation therapy to keep the irradiated field as small as possible may help reduce this risk. In patients who develop new dyspnea or atelectasis during osimertinib therapy without an apparent endobronchial mass on imaging, bronchoscopic evaluation should be considered.

## Patient perspective

The patient reported improvement in dyspnea and no longer required supplemental oxygen after treatment. She experienced no significant discomfort during management and was satisfied with the treatment outcome.

## Data Availability

The raw data supporting the conclusions of this article will be made available by the authors, without undue reservation.
