# Supplementary material for: Collagenous endobronchial obliteration after chemoradiation followed by consolidation osimertinib: a case report
Source: Front Oncol. 2026 Jul 31;16:1895328. doi: 10.3389/fonc.2026.1895328 (PMC13472905; doi:10.3389/fonc.2026.1895328)
Supplement: Supplementary file 1 [file Table1.docx]

- **CARE checklist of information to include when writing a case report**

| **Topic** | **Item** | **Checklist item description** | **Reported on Line** |
| --- | --- | --- | --- |
| **Title** | **1** | The diagnosis or intervention of primary focus followed by the words 'case report' | Title page |
| **Key Words** | **2** | 2 to 5 key words that identify diagnoses or interventions in this case report, including “case report” | Title page; 19-20 |
| **Abstract** | **3a** | Introduction: What is unique about this case and what does it add to the scientific literature? | Abstract; 23-26 |
| **Abstract** | **3b** | Main symptoms and/or important clinical findings | Abstract; 27-31 |
| **Abstract** | **3c** | The main diagnoses, therapeutic interventions, and outcomes | Abstract; 29-32 |
| **Abstract** | **3d** | Conclusion—What is the main “take-away” lesson(s) from this case? | Abstract; 38-42 |
| **Introduction** | **4** | One or two paragraphs summarizing why this case is unique **(may include references)** | Introduction; 46-55 |
| **Patient Information** | **5a** | De-identified patient specific information | Case presentation; 58-60 |
| **Patient Information** | **5b** | Primary concerns and symptoms of the patient | Case presentation; 79-80 |
| **Patient Information** | **5c** | Medical, family, and psycho-social history including relevant genetic information | Case presentation; 58-59 |
| **Patient Information** | **5d** | Relevant past interventions with outcomes | Case presentation; 60-78 |
| **Clinical Findings** | **6** | Significant physical examination and important clinical findings | Case presentation; 80-91 |
| **Timeline** | **7** | Describe significant physical examination (PE) and important clinical findings | Case presentation; 80-91 |
| **Diagnostic Assessment** | **8a** | Diagnostic testing (such as PE, laboratory testing, imaging, surveys) | Case presentation; 80-91 |
| **Diagnostic Assessment** | **8b** | Diagnostic challenges (such as access to testing, financial, or cultural) | N/A |
| **Diagnostic Assessment** | **8c** | Diagnosis (including other diagnoses considered) | Case presentation; 82-93 |
| **Diagnostic Assessment** | **8d** | Prognosis (such as staging in oncology) where applicable | Case presentation; 58-59 |
| **Therapeutic Intervention** | **9a** | Types of therapeutic intervention (such as pharmacologic, surgical, preventive, self-care) | Case presentation; 85-86 and 93-94 |
| **Therapeutic Intervention** | **9b** | Administration of therapeutic intervention (such as dosage, strength, duration) | Case presentation; 93-94 |
| **Therapeutic Intervention** | **9c** | Changes in therapeutic intervention (with rationale) | Case presentation; 93-101 |
| **Follow-up and Outcomes** | **10a** | Clinician and patient-assessed outcomes (if available) | Case presentation; 94-101 |
| **Follow-up and Outcomes** | **10b** | Important follow-up diagnostic and other test results | Case presentation; 97-103 |
| **Follow-up and Outcomes** | **10c** | Intervention adherence and tolerability (How was this assessed?) | Case presentation; 94-101 |
| **Follow-up and Outcomes** | **10d** | Adverse and unanticipated events | Case presentation; 97-101 |
| **Discussion** | **11a** | A scientific discussion of the strengths AND limitations associated with this case report | Discussion; 162-172 |
| **Discussion** | **11b** | Discussion of relevant medical literature **with references** | Discussion; 106-133 |
| **Discussion** | **11c** | Scientific rationale for conclusions (including assessment of possible causes) | Discussion; 134-161 |
| **Discussion** | **11d** | The primary “take-away” lessons of this case report (without references) in a one-paragraph conclusion | Conclusion; 175-181 |
| **Patient Perspective** | **12** | The patient should share their perspective in one to two paragraphs on the treatment(s) they received | Patient Perspective; 183-185 |
| **Informed Consent** | **13** | Did the patient give informed consent? Please provide if requested | **Yes** |
